# Identification and Functional Characterization of a Novel Long Non-Coding RNA NLRP14-OT in Turbot (*Scophthalmus maximus*) Immune Response Against *Vibrio anguillarum* Infection

**DOI:** 10.3390/ani16101443

**Published:** 2026-05-08

**Authors:** Beibei Wang, Yiying Liu, Yang Li, Xiaoli Liu, Ning Yang, Chao Li

**Affiliations:** School of Marine Science and Engineering, Qingdao Agricultural University, Qingdao 266109, China; w15288966538@163.com (B.W.); 15263348506@163.com (Y.L.); liyang13869986564@163.com (Y.L.); lxl17854231102@163.com (X.L.); yangning@qau.edu.cn (N.Y.)

**Keywords:** *Scophthalmus maximus*, lncRNA, inflammation, antibacterial

## Abstract

As an important member of non-coding RNAs, lncRNAs play a significant role in various biological processes, including regulating the immune response after bacterial infection. In this study, we identified a novel lncRNA (*NLRP14-OT*) in turbot (*Scophthalmus maximus*). As a result, *NLRP14-OT* was more than 2234 bp in length and was detectable in the liver, spleen, head kidney, intestine, skin, gill, muscles, blood, and brain, with the highest expression levels in the blood, followed by the spleen. To better understand the biological significance of lncRNAs in the immune system, this study performed qPCR on mucosal tissues (intestine, skin, and gill) at 0, 2, 6, 12, 24, and 48 h after *Vibrio anguillarum* (*V. anguillarum*) infection. Following infection with *V. anguillarum*, the expression of *NLRP14-OT* was significantly upregulated in the skin but significantly downregulated in the gill. Given that lncRNAs can regulate target genes through different mechanisms depending on their subcellular localization, we explored the localization of *NLRP14-OT*. Both RNA nucleoplasmic isolation and *FISH* experiments showed that *NLRP14-OT* was predominantly localized in the nucleus. GO and KEGG analyses indicated that *NLRP14-OT* was involved in several pathways related to the immune response such as the Tight junction, the Ras signaling pathway, and the Rap1 signaling pathway. Moreover, overexpression of *NLRP14-OT* in SMK cells significantly upregulated all tested immune genes, and this effect was further enhanced upon LPS stimulation at 6 h, suggesting a potential regulatory role for *NLRP14-OT* in the immune system. Meanwhile, it was found that overexpression of *NLRP14-OT* could activate the promoter activity of *NLRP14* gene and inhibit the NF-κB activity. The above results enrich the information of lncRNAs in teleost and provide a resource base for further research on the immune function of lncRNAs.

## Simple Summary

Farmed fish often suffer from bacterial diseases, which cause significant economic losses. Understanding how fish naturally fight infections can help develop better ways to protect them. In this study, we investigated a novel lncRNA, named *NLRP14-OT*, in turbot—a commonly farmed fish. This molecule does not code for a protein but instead helps regulate the fish’s immune system. We found that *NLRP14-OT* is present in many tissues, especially in the blood and spleen, which is important for immunity. When turbot was infected with a harmful bacterium, the levels of *NLRP14-OT* changed, indicating it plays a role in the defense response. Further experiments in SMK cells showed that increasing the amount of *NLRP14-OT* activated several immune-related genes, helping the cells respond to bacterial components. Our results demonstrate that *NLRP14-OT* acts as a positive regulator of the immune response in turbot. This finding provides new insights into how lncRNA molecules help fish fight infections.

## 1. Introduction

It has been confirmed that non-coding RNAs (ncRNAs) are functional regulators rather than “junk” transcripts, and they are found in cells ranging from yeast to vertebrates. Various cellular and physiological processes are mediated by these regulators [1,2]. Non-coding RNAs are typically classified into two categories based on length: long non-coding RNAs (lncRNAs), which exceed 200 base pairs, and small non-coding RNAs [3]. LncRNAs can be categorized as sense lncRNAs, antisense lncRNAs, bidirectional lncRNAs, intronic lncRNAs, and intergenic lncRNAs according to their positions on chromosomes related to their protein-coding genes [4]. Structurally, lncRNAs resemble mRNAs, consisting of a polyadenylation tail and a promoter structure after splicing. However, unlike mRNAs, they also typically contain a transcriptional stop codon. This means they cannot be translated into proteins [5,6]. Although lncRNAs are not directly involved in gene coding and protein synthesis, they play important roles as a class of endogenous ncRNAs in different physiological processes, including innate immunity [7,8,9]. LncRNAs have been reported to play a key role in maintaining cellular homeostasis and preventing the development of various diseases [10,11,12]. For example, lncRNA THRIL regulates TNF-α transcription by interacting with hnRNPL to control cytokine expression, prevents excessive inflammation, and alters the progression of inflammatory diseases [13]. Moreover, lncRNA DANCR has been identified as an important molecule involved in the pathophysiology of kidney injury, playing an essential role in the regulation of cell death and inflammatory cytokine production, which can be used for early detection and prediction of kidney problems [14].

In recent years, the role of lncRNAs in immune responses has been explored in several teleost. For instance, DE-lncRNAs involved in the regulation of host immune-related responses were identified in intestinal tissues of snakehead (*Channa argus*) after bacterial infection [15]. Linc-93.2 may regulate immune processes in common carp (*Cyprinus carpio*) macrophages by modulating NF-κB and estrogen receptor (ER) pathways [16]. In orange-spotted grouper (*Epinephelus coioides*), 105 lncRNAs were differentially expressed upon infection with Singapore grouper iridovirus (SGIV), and most of these differentially expressed lncRNAs were closely associated with regulation of immune-related genes [17]. In addition, Han et al. found that lnc432, as a ceRNA, reduced the inhibitory effect of miR-21-y on DAPK2, thereby enhancing the antibacterial ability of yellow catfish (*Pelteobagrus fuloidraco*) under hypoxic environments [18]. It was also found that lncRNAs mediate an antibacterial immune response in rainbow trout (*Oncorhynchus mykiss*) infected with *Flavobacterium psychrophilum* [19]. Meanwhile, lncRNA NARL, that was identified in miiuy croaker (*Miichthys miiuy*), can enhance NOD1-mediated antibacterial immune responses by interacting with miR-217-5p [20]. Despite the growing evidence indicating the presence of immune-related lncRNAs in aquatic animals, most of these studies have only identified differentially expressed lncRNAs in specific biological processes at the whole transcriptome level, and their molecular properties and functions remain far from thoroughly investigated.

The turbot (*Scophthalmus maximus*), introduced from the northeast Atlantic Ocean in 1992, has become one of the most important mariculture fishes in China due to its high economic value [21,22]. However, outbreaks of bacterial diseases, especially those caused by *Vibrio anguillarum* (*V. anguillarum*), pose great challenges to the industry [23,24,25]. Related studies have shown that lncRNA *SETD3-OT* was involved in the regulation of cell apoptosis and cycle, immune cell development, and the immune response against infection [26]; in response to LPS (a major component of the outer membrane of Gram-negative bacteria) stimulation, lncRNA *BCO1-AS* may increase the transcriptome and protein expression of caspase-1, therefore suppressing the production of inflammatory cytokines [27]. Transcriptome sequencing analysis of a *V. anguillarum*-infected turbot intestine and liver revealed that the target genes of DE-lncRNAs during infection were involved in a myriad of immune-related processes and showed significant correlations with immune-related signaling pathways [28,29]. The aim of this study was to determine the potential functions of a novel lncRNA named *NLRP14-OT*, which was identified as being involved in immune regulation in turbot. Subsequently, we analyzed the expression pattern of *NLRP14-OT* in turbot following infection with *V. anguillarum*. Furthermore, we investigated the response of SMK cells to *NLRP14-OT* after LPS treatment. This study highlights the positive regulatory role of *NLRP14-OT*, deepening our understanding of lncRNA functions in modulating immune responses in teleost.

## 2. Materials and Methods

### 2.1. Fish and Cells Lines

Turbot with an average body weight of 30 g and an average body length of 10.5 cm were purchased from an aquaculture company in Haiyang, Shandong Province, China. Prior to the experiment, the fish were acclimated for one week in a recirculating water system consisting of tanks with a capacity of 30 L filled with 20 L of recirculating seawater, at a stocking density of 15 fish per tank [28]. Turbot kidney cells (SMK) were maintained in Dulbecco’s Modified Eagle Medium (DMEM, Gibco, Paisley, SCT, UK), containing 10% FBS (Gibco, Grand Island, NY, USA), 100 μg/mL penicillin and streptomycin blend liquid (Solarbio, Beijing, China) at 24 °C in an incubator (Thermo Fisher Scientific, Asheville, NC, USA) and the human embryonic kidney (HEK) 293T cells were maintained in DMEM medium (Hyclone, Logan, UT, USA) supplemented with 10% FBS (Gibco, Grand Island, NY, USA), 100 μmL penicillin, and 100 g/mL streptomycin (Solarbio, Beijing, China) at 37 °C in an incubator (Thermo Fisher Scientific, Asheville, NC, USA) with 5% CO_2_.

### 2.2. Gene Cloning and Bioinformatics Analyses

Total RNA was extracted from mixed mucosal tissues, including intestine, skin, and gills, of healthy turbot using Trizol reagent (Takara, Shiga-ken, Japan) according to the manufacturer’s instructions. The extracted RNA was used to synthesize first-strand cDNA templates through Evo M-MLV 1st Strand cDNA Synthesis Kit (Accurate, Changsha, China) using the SMARTer RACE 5′/3′ Kit (TaKaRa, Shiga-ken, Japan) to perform 5′ and 3′ amplification via RACE experiments. Gene cloning was carried out with specific primers listed in Table 1. Subsequently, the RNA sequence of *NLRP14-OT* was submitted to the Coding Potential Calculator 2 (CPC2) and iLoc-LncRNA program to predict its coding potential and subcellular localization, respectively [30,31]. Finally, the *NLRP14-OT* gene sequence was preliminarily identified from the turbot genome database, and genomic localization analysis was performed.

### 2.3. Quantitative Real-Time PCR

To examine the tissue distribution of *NLRP14-OT* in turbot, all of these tissues were obtained independently from five healthy turbot, including head kidney, spleen, liver, skin, muscle, gill, intestine, blood and brain, and RNA from each organ/tissue was extracted as described above. For blood collection, approximately 1 mL of blood was drawn from the caudal vein of each turbot using a sterile syringe. The bacterial challenge was performed as described in previous studies [32,33]. In the challenge experiment, a total of 75 turbot were immersed in a solution of *V. anguillarum* with the concentration of 1 × 10^7^ CFU/mL for 2 h and then returned to recirculating tanks. Subsequently, intestine, skin, and gill were collected from 15 fish at each time point (2, 6, 12, 24, and 48 h post-immersion). In the control group, the *V. anguillarum* solution was replaced with sterilized LB medium, and the intestine, skin, and gill from 15 fish were collected for RNA extraction. Quantitative real-time PCR was performed in an Applied Biosystems QuantStudio 5 (Thermo Fisher Scientific, Asheville, NC, USA) using SYBR Green Premix Pro HS qPCR Kit (Accurate, Changsha, China). The amplification cycle was as follows: 95 °C for 30 s, 1 cycle; 95 °C for 5 s, 60 °C for 30 s, and 65 °C for 5 s, 40 cycles; then up to 95 °C at a rate of 0.1 °C/s increment. Triple biological replicates of RNA samples were prepared for gene expression analysis. The calculation of relative expression changes was performed using the 2^−ΔΔCt^ method [34]. 18S rRNA were employed as internal controls for lncRNA, respectively. The specific primers are listed in Table 1.

### 2.4. Plasmid Construction and Cell Transfection

For eukaryotic expression, the *NLRP14-OT* was amplified using PrimeSTAR Max DNA polymerase (Takara, Shiga-ken, Japan) with specific primers listed in Table 1 and inserted into pcDNA3.1 vector with his-tag by using the NheI and XhoI (Takara, Shiga-ken, Japan) restriction sites. To conduct dual-luciferase reporter assays, the promoters of turbot *NLRP14* were cloned with corresponding primers (Table 1) and inserted into the pGL3-basic luciferase reporter vector. All constructs were confirmed by sequencing to ensure the absence of unintended mutations. All transfections were performed using Lipofectamine 2000 (Invitrogen, Carlsbad, CA, USA). For stimulation experiments, after transfection of *NLRP14-OT* into cells for 24 h, LPS (10 μg/mL) and PGN (10 μg/mL) were incorporated into the cell culture medium. After 6 h of stimulation, cell samples were collected, and RNA was extracted using Trizol reagent to detect changes in the expression of certain genes.

### 2.5. Subcellular Localization and Fluorescence in Situ Hybridization (FISH) Assays

SMK cells (10^7^ cells per well) cultured at 24 °C were collected and washed three times with pre-cooled phosphate-buffered saline (PBS) to remove residual medium. Then, the SMK cells were subjected to cytoplasmic and nuclear RNA fractionation and purification using the PARIST kit (Thermo Fisher Scientific, Waltham, MA, USA). The quality and quantity of cytoplasmic and nuclear RNA were measured using the Nanodrop 2000 system (Thermo Fisher Scientific, West Palm Beach, FL, USA), and the OD260/280 ratios of all RNA samples were between 1.8 and 2.0, with OD260/230 ratios above 2.0, indicating high purity. Finally, the mRNA levels of *NLRP14-OT* in nuclear and cytoplasmic fractions were evaluated by qPCR assays. β-actin and U1 were used as cytoplasmic and nuclear controls, respectively. Digoxigenin-labeled RNA probes for *NLRP14-OT* were purchased from Sigma (Darmstadt, Germany). SMK cells were seeded in 24-well culture plate at 24 °C overnight and then fixed with 4% paraformaldehyde (Sigma, Darmstadt, Germany) for 30 min. The fixed cells were pre-incubated with pre-hybridization solution (Sigma, Darmstadt, Germany) at 70 °C for 4 h, followed by overnight hybridization with DIG-labeled probes at 70 °C. The cells were then blocked with 10% goat serum (Beyotime, Shanghai, China). Thereafter, cells were incubated with Cy^TM^3-conjugated IgG fraction monoclonal mouse anti-digoxin (Jackson ImmunoResearch, West Grove, PA, USA) for 1.5 h. The images were captured by fluorescence microscope (ZEISS, Jena, Germany).

### 2.6. Chromatin Isolation by RNA Purification and Mass Spectrometry (CHIRP-MS)

The ChIRP assay was performed using a Chromatin Isolation by RNA Purification (CHIRP) Kit (BersinBio, Guangzhou, China). Briefly, cell samples were crosslinked, followed by lysis on ice and sonication in an ice-bath until the sample became clear. Then, the cell lysate was incubated with agarose beads for 1 h to hybridize with the *NLRP14-OT* probe and the control probe. The mixture was then centrifuged to discard the supernatant and washed three times with PBS for pre-cleaning. Afterward, the samples were then denatured at 65 °C and hybridized overnight at 37 °C in hybridization buffer. Finally, the enriched DNA was extracted using the ethanol precipitation method and subjected to subsequent identification.

### 2.7. Dual-Luciferase Reporter Assays

To investigate the effects of *NLRP14-OT* on *NLRP14* promoter, HEK 293T cells were cultured in 24-cell plates at a density of 5 × 10^5^ cells/mL for 24 h and co-transfected with *NLRP14-OT* (or empty vector as a control), the target promoter–luciferase plasmid (p*NLRP14*pro-Luc) and pRL-TK. At 6 h post-transfection, the culture medium was renewed. At 24 h post-transfection, luciferase activity was measured using a Dual-Luciferase Reporter Assay Kit (Vazyme, Nanjing, China). Similarly, to analyze the functional regulation of *NLRP14-OT* or *NLRP14*, HEK 293T cells were co-transfected with either *NLRP14-OT* expression plasmid or *NLRP14* expression plasmid, together with NF-κB luciferase reporter gene plasmids, pRL-TK Renilla luciferase plasmid, either empty vector as a control. All luciferase activity values were normalized to Renilla luciferase activity as an internal standard.

### 2.8. Statistical Analysis

Each experiment included at least three biological replicates. In all experiments, differences between the treatment group and the control group were analyzed using Student’s t-test in SPSS software (v.20.0), with a significance level of *p* < 0.05. Data were presented as means ± standard error (SE).

## 3. Results

### 3.1. Characterization of NLRP14-OT

**Figure 1:**
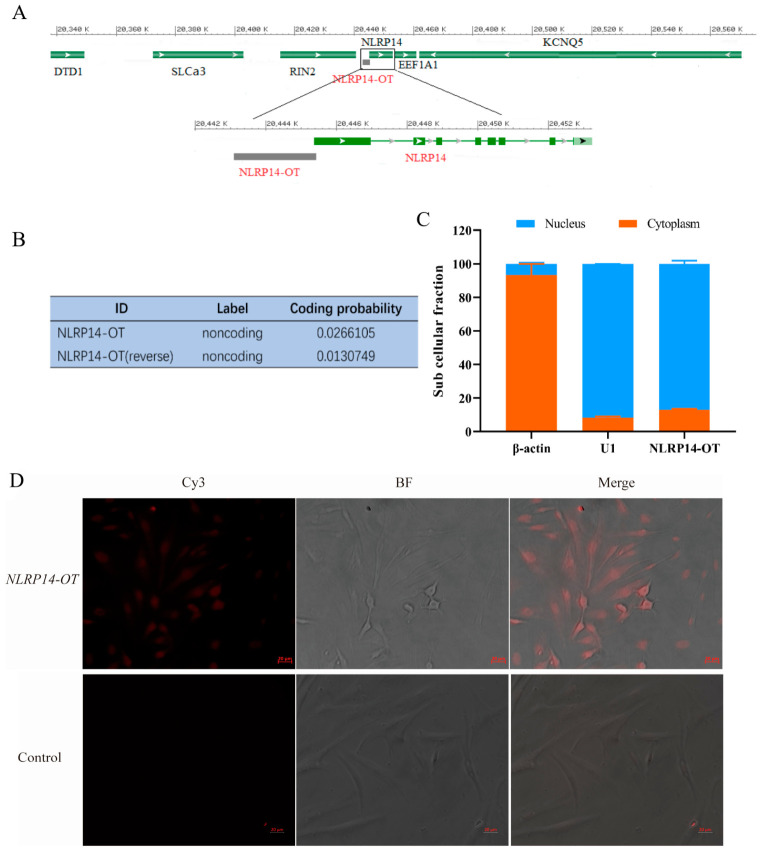
Characterization of *NLRP14-OT.* (**A**) The genomic location of *NLRP14-OT* and its neighboring genes in the genome location within 100 kbp. (**B**) *NLRP14-OT* was predicted to be non-coding RNA. The RNA sequences of *NLRP14-OT* were put into the Coding Potential Calculator (CPC) program, which was predicted to be non-coding RNAs. (**C**) Isolated nucleoplasmic RNA experiments showing that *NLRP14-OT* was mainly localized in the nucleus (*n* = 3 independent biological replicates). (**D**) *FISH* staining showing that *NLRP14-OT* was mainly localized in the nucleus. Red fluorescence (Cy3) represents *NLRP14-OT*, the bright field (BF) indicates cells, and the Merge image on the right shows the combination of red fluorescence and bright field. Bar = 20 μm.

The full length of *NLRP14-OT* was 2234 bp, containing a poly(A) tail and located on chromosome 8 (Figure 1A). Subcellular fractionation experiments showed that the cytoplasmic marker β-actin was predominantly detected in the cytoplasm 93.34%, while the nuclear marker U1 was predominantly detected in the nucleus 91.81%, confirming the efficiency of the fractionation. Under these conditions, *NLRP14-OT* was found to be mainly localized in the nucleus, with 86.97% in the nuclear fraction and 13.03% in the cytoplasmic fraction (Figure 1B). Next, the subcellular localization of *NLRP14-OT* in SMK cells was further confirmed by fluorescence in situ hybridization (*FISH*) assay. The red fluorescence signals were predominantly observed in the nuclear region, while the cytoplasm showed only weak or no signal, further confirming that *NLRP14-OT* was mainly localized in the nucleus (Figure 1C). The RNA sequence of lncRNA *NLRP14-OT* was put into the Coding Potential Calculator (CPC) program, which was predicted to be non-coding RNA (Figure 1D).

### 3.2. Tissue Distribution of NLRP14-OT

**Figure 2:**
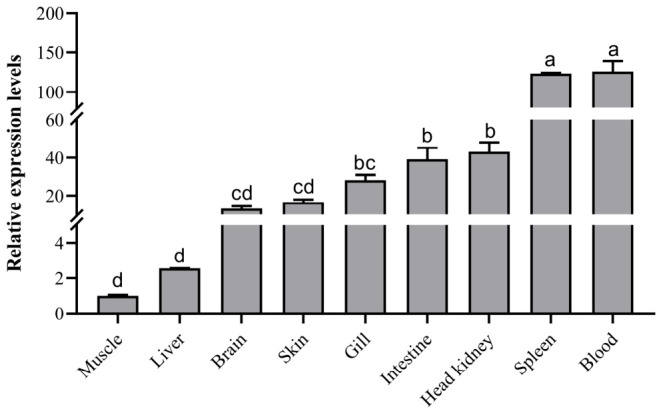
Tissue distribution of *NLRP14-OT* in different healthy tissues of turbot. Expression levels were calibrated against tissue that had the lowest expression level, and *18S rRNA* was used as a reference gene. All data represented the mean ± SE from three independent triplicated experiments. Different letters indicate statistically significant differences.

The expression patterns of *NLRP14-OT* in nine examined tissues of *S. maximus* were measured by qPCR. The analysis revealed that *NLRP14-OT* was expressed widely in all tested tissues/organs of turbot, especially in the blood that showed the highest expression levels of *NLRP14-OT*, which was followed by the spleen, head kidney and intestine (Figure 2). In those traditional immune tissues (spleen and kidney), the expression level of *NLRP14-OT* reached 123.3 and 43.1 times that of the muscle, respectively, and a 39.1-fold expression level was also observed in intestine.

### 3.3. Expression of NLRP14-OT Post-Infection

**Figure 3:**
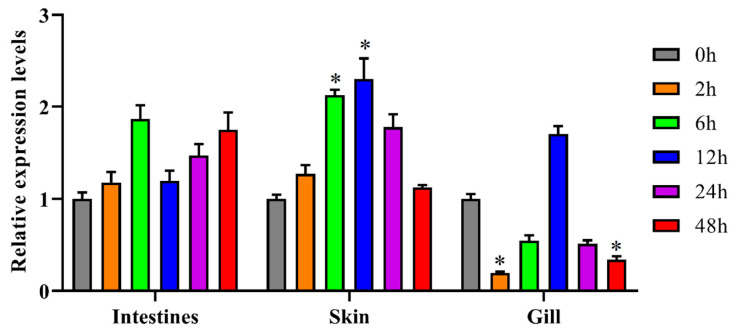
The mRNA expressions of *NLRP14-OT* after *Vibrio anguillarum* (*V. anguillarum*)infection. lncRNA *NLRP14-OT* expression profiles in turbot intestine, skin and gill 2, 6, 12, 24 and 48 h after *V. anguillarum* infection. Expression level was calculated by the change in expression at a given time point relative to the untreated control and normalized by change in the *18S rRNA* housekeeping gene. All data represented the mean ± SE from three independent triplicated experiments and significant difference was indicated by * *p* < 0.05.

Quantitative real-time PCR results revealed that *NLRP14-OT* expression exhibited an overall upregulation trend in intestine following *V. anguillarum* infection; in skin, *NLRP14-OT* expression was significantly upregulated during infection, particularly at 6 h and 12 h post-infection (2.12-fold and 2.3-fold, respectively). However, in gill, *NLRP14-OT* expression was significantly downregulated by 8.1-fold within 2 h post-infection and by 6.6-fold within 48 h (Figure 3).

### 3.4. Expression Patterns of NLRP14-OT Transcripts in Response to Various PAMPs

**Figure 4:**
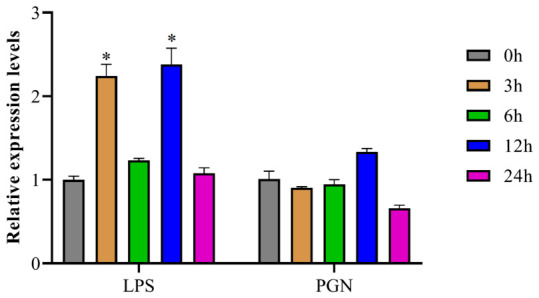
Results of the in vitro stimulation of *BCO1-AS* in responses to LPS and PGN in SMK cells. All data represents the mean ± SE from three independent triplicated experiments and significant differences are indicated by * *p* < 0.05.

In order to gain a deeper understanding of the expression dynamics of *NLRP14-OT* during immune activation, we used qRT-PCR to detect its mRNA levels in the SMK cells following stimulation with LPS and PGN to investigate its role in immune system activation. As shown in Figure 4, in LPS-stimulated SMK cells, *NLRP14-OT* expression showed an overall upward trend, with a 2.24-fold increase at 3 h and a 2.38-fold increase at 12 h, but the level of *NLRP14-OT* had no such significant change in PGN challenge.

### 3.5. The Potential Immune Signaling Pathways Involving NLRP14-OT Detected by CHIRP

**Figure 5:**
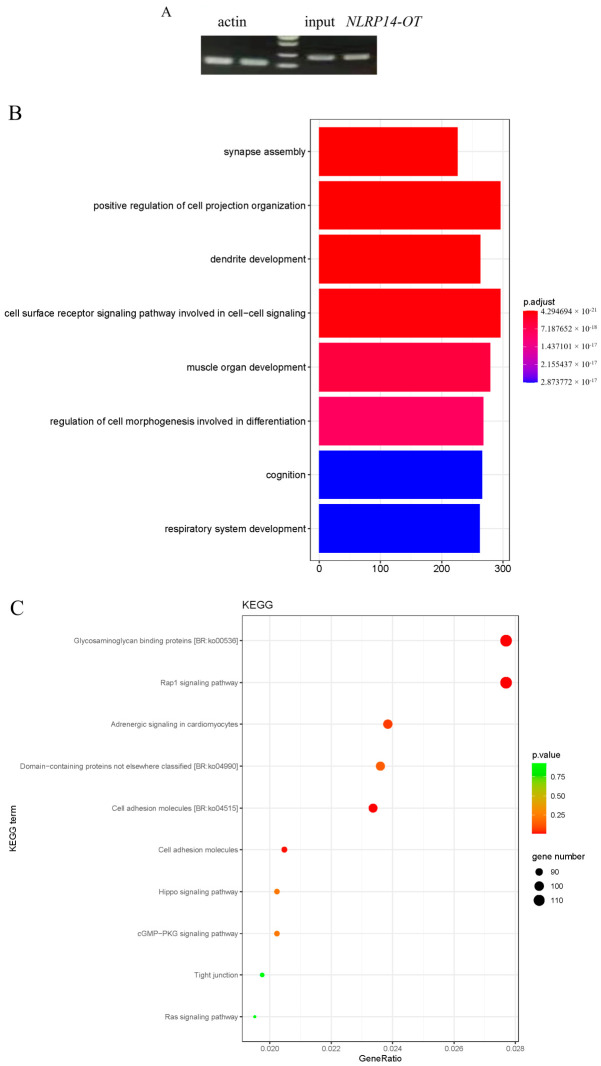
*NLRP14-OT* was involved in regulating several potential signaling pathways of the immune system. (**A**) ChIRP enriched for RNA. (**B**) GO enrichment of lncRNA-related function. (**C**) The ten most significant signaling pathways in *NLRP14-OT* KEGG enrichment analysis. Statistical significance is indicated by different colors. The colorful bar refers to the q-value of the respective signaling pathway. The size of the circle c genes in each pathway.

ChIRP-qPCR assays showed that the probes targeting *NLRP14-OT* significantly enriched *NLRP14-OT* RNA compared to the control probes (Figure 5A). Sequencing analysis of the enriched DNA fragments identified multiple genomic regions interacting with *NLRP14-OT*, including those corresponding to immune-related genes such as *NLRP12*, *IL-10R*, and *caspase-1* (Table 2). Additional interacting regions were associated with sodium channel protein-mediated ion transport. GO enrichment analysis revealed eight significantly enriched biological process terms, including “synapse assembly”, “muscle organ development”, and “regulation of cell morphogenesis involved in differentiation” (Figure 5B). KEGG pathway analysis showed that the genes potentially interacting with *NLRP14-OT* were enriched in several signaling pathways, including the Rap1 signaling pathway, the Ras signaling pathway, and the Tight junction pathway (Figure 5C).

### 3.6. NLRP14-OT Enhances the Expression of Inflammatory Cytokines

**Figure 6:**
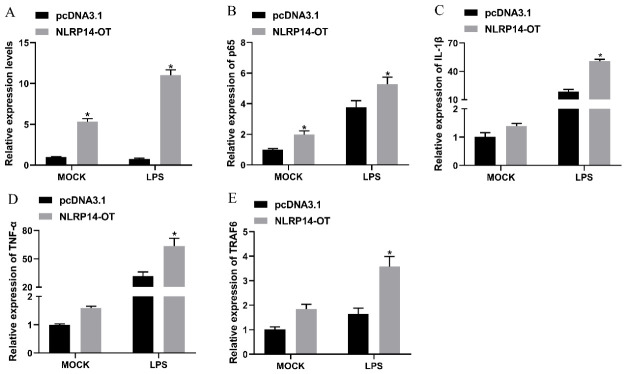
*NLRP14-OT* enhances the expression of inflammatory cytokines. (**A**) SMK cells were transfected with *NLRP14-OT* plasmid and pcDNA3.1 vector for 24 h. Then, the cells were treated with LPS for 6 h and the expression of *NLRP14-OT* was determined by qPCR and normalized to *18s rRNA*. (**B**–**E**) Following transfection with the *NLRP14-OT* expression plasmid for 24 h, the gene expressions of *p65* (**B**), *IL-1β* (**C**), *TNF-α* (**D**) and *TRAF6* (**E**) in the SMK cells were stimulated by LPS for 6 h. All data represents the mean ± SE from three independent triplicated experiments and significant difference is indicated by * *p* < 0.05.

As *NLRP14-OT* expression increased significantly following *V. anguillarum* and LPS stimulation, we speculated that *NLRP14-OT* might be associated with the signaling pathway induced by Gram-negative bacteria and LPS stimulation. To probe the functions of *NLRP14-OT* in the LPS stimulation inflammatory response, a full-length expression plasmid of *NLRP14-OT* was constructed. First, we transfected *NLRP14-OT* and pcDNA3.1 into SMK cells and detected the expression of *NLRP14-OT*, *p65*, *IL-1β*, *TNF-α* and *TRAF6* before and after LPS stimulation. The result showed that the expression plasmid of *NLRP14-OT* can efficiently increase the expression of *NLRP14-OT* (Figure 6A). Figure 6B–E show that overexpression of *NLRP14-OT* greatly promoted the expression levels of *p65*, *IL-1β*, *TNF-α* and *TRAF6* in response to LPS stimulation. This indicated that *NLRP14-OT* can promote the antimicrobial response and increase the expression of inflammatory cytokines.

### 3.7. NLRP14-OT Positively Regulates NLRP14-Mediated Immune Pathways

**Figure 7:**
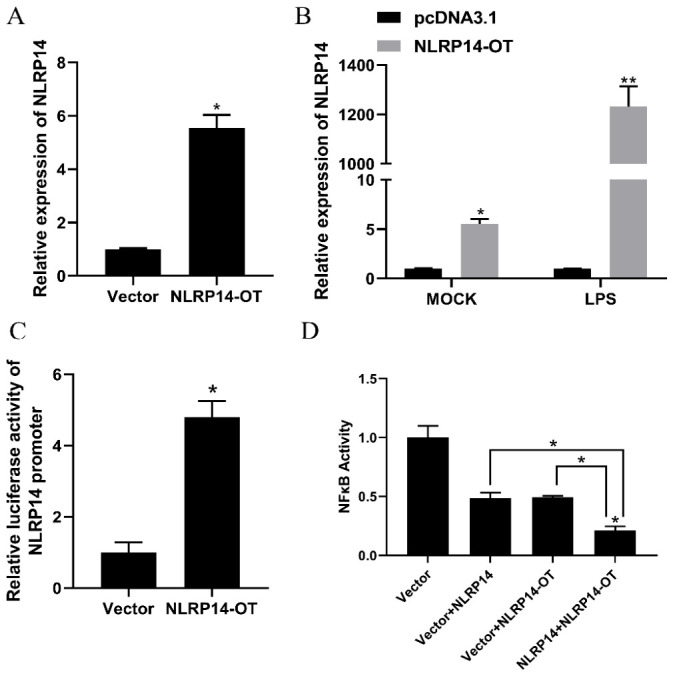
*NLRP14-OT* positively regulates *NLRP14*-mediated immune pathways. (**A**) SMK cells were co-transfected with pcDNA3.1 vector or *NLRP14-OT* expression plasmid, and the expression of *NLRP14* was detected by qPCR 24 h later. (**B**) The mRNA levels of *NLRP14* in SMK cells stimulated by LPS were determined by qPCR normalized to β-actin. (**C**) The *NLRP14-OT* expression plasmid and *NLRP14* promoter together with the pRL-TK Renilla luciferase plasmid were co-transfected with HEK 293T cells for 24 h. Then the cells were collected and the luciferase activity was assessed. (**D**) HEK 293T cells were co-transfected with NF-κB-luc (450 ng), pRL-TK (100 ng), together with *NLRP14-OT* (225 ng) and *NLRP14* 225 ng alone or in combination. The pcDNA3.1 empty vector was used to balance the total volume of transfected plasmids for 1000 ng. At 24 h post-transfection, cells were collected to detect luciferase activity. All data represents the mean ± SE from three independent triplicated experiments and significant difference is indicated by * *p* < 0.05 and ** *p* < 0.01.

*NLRP14-OT* has been found to encourage the production of inflammatory substances and overlap 90 bp with the cDNA sequence of *NLRP14*; the *NLRP14-OT* plasmid was transfected into SMK cells for 24 h; and the expression of *NLRP14* was detected by qPCR. As demonstrated in Figure 7A, in comparison with the control group (untreated cells), the expression of *NLRP14-OT* can substantially enhance the expression of *NLRP14*. Following the stimulation of LPS in *NLRP14-OT* overexpressed cells, there was a dramatic increase in the expression of *NLRP14* to 1231.8-fold expression levels at 6 h post-stimulation (Figure 7B). These results indicated that *NLRP14-OT* can positively regulate *NLRP14* at the level of mRNA. To understand whether *NLRP14-OT* is involved in *NLRP14*-mediated immune signaling, promoter reporter plasmids of *NLRP14* were co-transfected with expression plasmids of *NLRP14-OT* in HEK 293T cells. It was observed that overexpression of *NLRP14-OT* induced a significantly higher level of *NLRP14* promoter activity compared with the control group (Figure 7C). Luciferase assays showed that the overexpression of *NLRP14* alone decreased NF-κB activity, while co-expression of *NLRP14* and *NLRP14-OT* induced a significantly lower level of NF-κB activity when compared with the single overexpression of *NLRP14* (Figure 7D).

## 4. Discussion

lncRNAs, as important non-coding RNA molecules, have been detected to play key roles in a variety of biological processes including immune response, growth and development [35,36]. Previous studies have demonstrated that lncRNAs participate in various immune responses in vertebrates, including multiple pathways associated with innate and adaptive immunity [37,38,39], as well as certain immune-mediated diseases [40,41]. For example, the lncRNA PACER has been shown to bind to the inhibitory NF-κB subunit p50, preventing it from exerting its suppressive effect on the COX-2 promoter region. This may promote the formation and function of the active NF-κB p65/p50 dimer [42]. As a key positive regulator of the innate immune response in miiuy croaker, NARL plays a critical role of inhibiting the feedback of the NOD1-NF-κB/IRF3-mediated signaling pathway [20]. In our study, we discovered that lncRNA *NLRP14-OT* positively regulates the immune response in turbot.

To gain deeper insight into the specific function of an individual lncRNA, subcellular localization analysis was often employed, as it can provide valuable information. LncRNAs in the nucleus may exert their regulatory functions through interactions with nuclear proteins, whereas those in the cytoplasm primarily function by modulating mRNA translational stability [43,44]. In line with the expected findings, our investigation showed that *NLRP14-OT* was mostly found inside the cellular nucleus.

The expression of the newly identified lncRNA *NLRP14-OT* was examined by qPCR in nine tissues of healthy turbot. The results indicate that *NLRP14-OT* exhibits constitutively high expression levels in the blood, spleen, head kidney, intestine, and gill. The highest expression was observed in the blood, suggesting that it may play a regulatory role in the immune system via the vascular system. Furthermore, the high expression of *NLRP14-OT* in classical immune tissues (spleen and kidney) reflects its potential involvement in systemic immune surveillance and pathogen clearance, as these organs are central to hematopoiesis, antigen presentation, and immune cell activation in teleosts [45]. In mucosal tissues such as the intestine, skin, and gill, the abundant expression of *NLRP14-OT* suggests a role in local mucosal immunity.

In recent years, numerous studies had revealed that lncRNAs play an indispensable role in immune responses to various bacterial, viral, or parasitic infections in multiple teleost species, such as Atlantic salmon (*Salmon salar*) [46,47], Nile tilapia (*Oreochromis nilotica*) [48], snakehead [15], koi carp [49], large yellow croaker (*Larimichthys crocea*) [50] and coho salmon (*Oncorhynchus kisutch*) [51]. In order to investigate the key functions of *NLRP14-OT* in antibacterial immunity, the expression levels of the *NLRP14-OT* gene were examined in turbot mucosal tissues following *V. anguillarum* infection in vivo, as well as in SMK cells stimulated with LPS and PGN in vitro. In this study, the expression of *NLRP14-OT* in turbot gills was inhibited, while the expression of *NLRP14-OT* in skin was increased with the prolongation of *V. anguillarum* infection. Meanwhile, *NLRP14-OT* was markedly upregulated in SMK cells, showing an increasing trend during LPS stimulation. These results suggest that *NLRP14-OT* might play a crucial role in fish defense against bacterial infections by being activated through a pathogen-ethical immune response. Moreover, previous research has documented the immune functions of lncRNA in inflammation in turbot [52].

The NF-κB pathway, as one of the essential signaling pathways regulating inflammatory responses, can rapidly and transiently activate transcriptional effects [53]. In this study, overexpression of *NLRP14-OT* significantly increased the mRNA expression levels of *p65*, *IL-1β*, *TNF-α*, and *TRAF6* in SMK cells, indicating that overexpression of *NLRP14-OT* might induce an inflammatory response in turbot by activating the NF-κB signaling pathway. Similarly, overexpression of lncRNA has been found to markedly increase the expression levels of inflammation-related genes in teleost fish, including miiuy croaker [54], common carp [16] and grass carp (*Ctenopharyngodon idella*) [55]. Notably, in olive flounder (*Paralichthys olivaceus*), the expression trend of NF-κB was not consistent with that of its downstream inflammation-related genes, suggesting that NF-κB may be activated post-transcriptionally or regulated by other transcription factors, which is needed to elucidate the underlying mechanisms [56,57,58]. In this study, we found a significant association between *NLRP14-OT* and NF-κB signaling pathway-related genes in turbot immune tissues. Under LPS stimulation, *NLRP14-OT* and NF-κB signaling pathways appear to be cooperatively activated to enhance immune defense. This provides new insights into fish immune responses. Future research may require characterization of the mutual regulation and correlation between them.

Several subfamilies exist within the NLR protein family, distinguished by their different N-terminal effector domains [59]. Nucleotide-binding oligomerization domain (NOD)-like receptors with a pyrin domain (PYD), NLRP subfamily (also known as the NALP family), are a newly identified NLR group [60]. Previous studies have indicated that NLRP proteins participate in apoptosis and inflammatory signaling pathways by forming large signaling platforms (named inflammasomes) and activating caspase activity, thereby playing key role in innate immunity [61,62,63]. *NLRP14* is a member of the NLRP subfamily that is specifically expressed in the gonads, and research on it remains limited. Overexpression of *NLRP14* in HEK 293T cells was found to inhibit numerous innate immune signaling pathways. Conversely, suppression of *NLRP14* expression enhanced nucleic acid sensing pathways in response to agonists such as double-stranded DNA [64]. The present study showed that *NLRP14-OT* significantly induced the activity of *NLRP14* promoter and, upon co-transfection, significantly inhibited the activity of the NF-κB signaling pathway, which is consistent with the findings described above.

## 5. Conclusions

In summary, we identified a novel lncRNA, *NLRP14-OT*, which is 2234 bp in length. It was mainly expressed in the blood and spleen of turbot. Following infection of turbot by *V. anguillarum*, the expression of *NLRP14-OT* was changed, suggesting its involvement in regulating the inflammatory response, and the exposure of SMK cells to LPS suggested *NLRP14-OT* involvement in the immunoregulatory effects. Furthermore, overexpression of *NLRP14-OT* enhanced the production of inflammatory cytokines. Finally, we confirmed that *NLRP14-OT* significantly increased the expression of *NLRP14* and induced markedly higher *NLRP14* promoter activity. Overall, this study provides new insights into the role of lncRNAs in fish and offers a novel perspective on mucosal immune defense against bacterial invasion in teleost.

## Figures and Tables

**Table 1:** Primers used in this study.

Primer	Sequence (5′ to 3′)
5′RACE-R1	CTCCACGAAGACACTTTTGAATGC
5′RACE-R2	CGCTTGAAAGCCGTTTTGTTTG
3′RACE-F1	GAAAATAAGGAAATACGCTCATCGG
3′RACE-F2	CAAAATTCCCTGATGTCAGCGTCT
Full-F	ACTAGTGTCTCGTCTCCAGACCCG
Full-R	TAAAATATGCTTCATTCAGTTGCTGATA
q*NLRP14-OT*-F	CCTCGTTCTTCTCGGTAAGT
q*NLRP14-OT*-R	GGTGGTGCGTGGTTTCGTTT
qTNF-α-F	GGGTGGATGTGGAAGGTGAT
qTNF-α-R	GGCCTCTGTTTGGCTTGACT
qIL-1β-F	TGGAGAGCATCGTGGAAGAAC
qIL-1β-R	CGCCCGTCCTGCTGAAC
qTRAF6-F	AGAGATGCCCTGTGGACAAC
qTRAF6-R	CGCCAAATGATTCTCCAGTT
qNF-κB-P65-F	ATGCCTTTGAGGACCTTTT
qNF-κB-P65-R	GTGTTCTGGGATGCTGTGT
18S-F	TGTGGGTTTTCTCTCTCTG
18S-R	ATTCTTGGCAAATGCTTTC
β-actin-F	GTAGGTGATGAAGCCCAGAGCA
β-actin-R	CTGGGTCATCTTCTCCCTGTTG
*U1*-F	GAACGCAGTCCCCCACTAC
*U1*-R	TACTTACCTGGCAGGGGAGATAC
pcDNA3.1-*NLRP14-OT*-F	AGACCCAAGCTGGCTAGCACTAGTGTCTCGTCTCCAGACC
pcDNA3.1-*NLRP14-OT*-R	GGGCCCTCTAGACTCGAGCTAATGATGATGATGATGATGATGATGTAAAATATGCTTCATTCAGTTGCT

Note: Primers for *18S rRNA*, *TNF-α*, *IL-1β*, *U1* and *β-actin* were from our previous studies [27,32]. All other primers were newly designed for this study.

**Table 2:** Identification of *NLRP14-OT* partially interacting DNAs. Table shows the identity of lncRNA *NLRP14-OT* interacting DNAs obtained by ChIRP-seq.

PeakID	Chr	Gene Description
S230_peak_10382	NC_049705.1	Rac GTPase-activating protein 1
S230_peak_5216	NC_049696.1	Dedicator of cytokinesis 5
S230_peak_11641	NC_049709.1	Natriuretic peptide receptor 1a
S230_peak_1596	NC_049690.1	Caspase-1-A-like
S230_peak_7943	NC_049701.1	Interleukin-10 receptor like
S230_peak_6121	NC_049697.1	NLR family CARD domain containing 5
S230_peak_1863	NC_049690.1	Scavenger receptor cysteine-rich type 1 protein M130
S230_peak_3322	NC_049693.1	NACHT LRR and PYD domains-containing 12-like
S230_peak_9442	NC_049704.1	Exocyst complex component 3-like protein 4
S230_peak_10838	NC_049706.1	Sodium channel voltage-gated

## Data Availability

The original contributions presented in this study are incorporated within the article. Further enquiries should be directed to the corresponding author.

## Abbreviations

lncRNA	long non-coding RNA
miRNAs	microRNAs
ceRNA	competitive endogenous RNA
CFU	colony-forming units
bp	base pair
cDNA	complementary DNA
RACE	rapid amplification of cDNA end
IL-1β	interleukin 1 beta
TNF-α	tumor necrosis factor alpha
TRAF6	tumor necrosis factor receptor-associated factor 6
LPS	lipopolysaccharide
PGN	peptidoglycan
PCR	polymerase chain reaction
NF-κB	nuclear factor kappa B
